# The role of the retinoblastoma protein-interacting zinc finger gene 1 tumor suppressor gene in human esophageal squamous cell carcinoma cells

**DOI:** 10.3892/ol.2013.1608

**Published:** 2013-10-09

**Authors:** SHANGWEN DONG, PENG ZHANG, SHAOJIE LIANG, SHUO WANG, PEI SUN, YUANGUO WANG

**Affiliations:** 1 Department of Cardiothoracic Surgery, Tianjin Medical University General Hospital, Heping, Tianjin 300052, P.R. China; 2 Tianjin Institute of Endocrinology, Tianjin Medical University, Heping, Tianjin 300070, P.R. China

**Keywords:** esophageal squamous cell carcinoma, retinoblastoma protein-interacting zinc finger gene 1, transfection, pcDNA3.1(+), 5-aza-2′-deoxycytidine

## Abstract

The tumor suppressor protein retinoblastoma protein-interacting zinc finger gene 1 (RIZ1) is downregulated in several types of cancer, including esophageal squamous cell carcinoma (ESCC). The present study used two *in vitro* methods to re-express RIZ1 in the human ESCC TE13 cell line in order to induce apoptosis. RIZ1 was re-expressed in the TE13 cells by reintroducing the gene through transfection or by removal of transcriptional repression through treatment with a DNA methyltransferase (DNMT) inhibitor. To reintroduce the gene, the open reading frame of the RIZ1 gene was inserted into the eukaryotic expression pcDNA3.1(+) vector and pcDNA3.1(+)/RIZ1 was purified and transfected into the TE13 ESCC cells. Removing transcriptional repression involved treating the TE13 cells with 5-aza-2′-deoxycytidine (5-aza-CdR), a DNMT inhibitor. RIZ1 mRNA and protein expression were determined by quantitative polymerase chain reaction (qPCR) and western blotting. The rate of apoptosis of the cells was determined by flow cytometry. A recombinant eukaryotic human RIZ1 expression plasmid, pcDNA3.1(+)/RIZ1, was constructed and confirmed by sequencing. RIZ1 mRNA and protein expression increased in pcDNA3.1(+)/RIZ1 stably transfected cells. Treatment with 5-aza-CdR for 48 and 72 h resulted in increased RIZ1 protein expression and increased the rate of apoptosis in the TE13 cells (P<0.01). In conclusion, transfection of the TE13 cells with the eukaryotic pcDNA3.1(+)/RIZ1 expression vector and reversal of transcriptional repression of RIZ1 using 5-aza-CdR demonstrate that it is possible to re-express RIZ1 in TE13 cells. Furthermore, the re-expression of RIZ1 led to an increased rate of apoptosis and this method may provide new therapeutic possibilities.

## Introduction

Esophageal squamous cell carcinoma (ESCC) is a malignancy that arises from esophageal epithelial cells and represents ~2% of all tumor types by incidence (1). The treatment for esophageal cancer includes surgery, radiotherapy and chemotherapy. Early to middle stage esophageal cancer is often curable, with late stage disease having a poor prognosis (2). Despite advances in technology and an improvement in the survival rate for esophageal cancer, the efficacy of treatment remains far from satisfactory. The main reasons for treatment failure include a change in respiratory and digestive function following surgery, and the damage and side effects that are associated with chemotherapy (1). Consequently, new methods for the treatment of early and late stage disease are required.

Retinoblastoma protein-interacting zinc finger gene 1 (RIZ1) plays a significant role as a tumor suppressor gene in esophageal cancer. RIZ1 has previously been reported to be expressed at low levels in esophageal carcinoma tissues compared with the adjacent non-cancerous tissues (3,4). Furthermore, the expression of RIZ1 may also be regulated by methylation of the gene promoter (4). The ESCC TE13 cell line was selected, which expresses low levels of RIZ1, to confirm the existence of methylation in the RIZ1 promoter in ESCC cells. To study the tumor suppressor role of RIZ1, the TE13 cells were treated with a DNA methyltransferase (DNMT) inhibitor, 5-aza-2′-deoxycytidine (5-aza-CdR), in order to reverse the methylation of the RIZ1 promoter and re-express the protein. Furthermore, a eukaryotic vector was constructed, which expressed human RIZ1. The effects of re-expressing RIZ1 using the vector or by treatment with 5-aza-CdR on apoptosis were investigated in the TE13 cells. The present study aimed to identify a new therapeutic target and provide a foundation for gene therapy in esophageal cancer.

## Materials and methods

The study was conducted in accordance with the Declaration of Helsinki. Approval for this study was obtained from the Ethics Committee for the Use of Human Subjects of Tianjin Medical University General Hospital (Tianjin, China). Patients provided their written consent to participate in this study and this consent was also approved by the Ethics Committee for the Use of Human Subjects of Tianjin Medical University General Hospital.

### Cell lines and tissue samples

The human ESCC TE13 cell line was purchased from American Type Culture Collection (Rockville, MD, USA) and cultured in RPMI-1640 containing 4.76 g HEPES, 2.0 g NaCO_3_, 10.4 g RPMI-1640 and 1,000 ml ddH_2_O (Gibco, Carlsbad, CA, USA), supplemented with 10% fetal bovine serum (Gibco), 1X L-glutamine (2 mM), 100 U/ml penicillin and 100 μg/ml streptomycin. The cells were incubated at 37°C in a 5% CO_2_ humidified incubator.

The esophageal cancer tissues and the matched adjacent non-cancerous tissue samples were obtained from the Department of Cardiothoracic Surgery of Tianjin Medical University General Hospital following the surgical excision of the tumors. All the specimens were placed in liquid nitrogen immediately following the resection and stored at −80°C until RNA or genomic DNA extraction. None of the patients were administered chemotherapy or radiation therapy prior to surgery and the diagnoses of all the patients were pathologically confirmed to be esophageal squamous carcinoma.

### Isolation of RNA from cell lines and tissue samples

RNA was isolated from cell lines or tissues using TRIzol (Invitrogen, Carlsbad, CA, USA) according to the manufacturer’s instructions. TRIzol (1 ml) was added to 5×10^6^-1×10^7^ cultured cells and the tissue samples were ground into a fine powder using a pestle and mortar prior to incubation in TRIzol (100 g/l). The RNA pellets were resuspended in diethylpyrocarbonate-treated H_2_O. The total RNA concentrations of the samples were quantified using a UV spectrophotometer (DU-460, Beckman Coulter, Miami, FL, USA).

### Reverse transcription amplification of RIZ1 mRNA

Reverse transcription was performed to produce complementary DNA (cDNA) using 2 μg RNA, molony murine leukemia virus (M-MLV) reverse transcriptase, ribonuclease inhibitor and dNTPs mixture (Takara Bio, Inc., Shiga, Japan) according to the manufacturer’s instructions. Semi-quantitative polymerase chain reaction (PCR) was performed using the cDNA templates.

According to the published NCBI RIZ1 mRNA sequence (NM_012231), the size of the protein coding region is 5,157 base pairs that are positioned between base pairs 857 and 6,013. Due to the size of the amplicon, the open reading frame may be divided into five sections, designated A603, A1200, B, C and D. The primers were previously designed for the five amplicons of RIZ1 by Primer 5.0 software (Premier, Palo Alto, CA, USA) as follows: Forward: 5′-GTGGCTAGCATGAATCAGAACACTACTG-3′ and reverse: 5′-TTGGCCAGAGGTGAAATCTGG CTC-3′ for A603; forward: 5′-TGGCTGCGATATGTGA ATTG-3′ and reverse: 5′-CTCTACGCTGATGCCGTCTC-3′ for A1200; forward: 5′-GCTGATGGCAAAGCATCTG-3′ and reverse: 5′-AATTCCTTGCCTTCAGAGTCAC-3′ for B; forward: 5′-TCAAAGAAAGTCATTCAGTGC-3′ and reverse: 5′-CGGTGATGGTACTGAAATG-3′ for C; and forward: 5′-GCCTCAATCAGCATTACC-3′ and reverse: 5′-GTCTACTCTTTGAAGAATGGTC-3′ for D. PCR amplification for each amplicon (A603, A1200, B, C and D) of RIZ1 was also performed using cDNA from normal, control esophageal tissue. The PCR reactions were performed in a volume of 50 μl, consisting of 5 μl 10X KOD buffer, 5 μl 2 M dNTPs, 3 μl 25 mM MgSO_4_, 2 μl each of the forward and reverse primers, 1 μl cDNA, 1 μl KOD-Plus Ver. 2 enzyme (Toyobo Co., Ltd., Osaka, Japan) and ddH_2_O. Each PCR amplification required specific conditions according to the melting temperature and size of the amplicon as follows: Initialization at 94°C for 2 min, 35 cycles of denaturation at 98°C for 10 sec, annealing (A603, 60°C at 30 sec; A1200, 57°C at 30 sec; B, 55°C at 30 sec; C, 50°C at 30 sec and D, 50°C at 30 sec), amplification at 72°C 1 min and a final extension at 72°C for 10 min. The quality of the amplified products was analyzed using 12 g/l agarose gels with a UV spectrophotometer (Beckman Coulter) and the quantitative PCR reaction products were sequenced.

### Construction of pcDNA3.1(+)/RIZ1

The amplicons were extracted from the agarose gel using the TIANgel Midi Purification kit (Tiangen Biotech Co., Ltd., Beijing, China) according to the manufacturer’s instructions. Each of the five amplicons were separately inserted into a T Trans1-T1 Phage Resistant vector (Promega Biotech Co., Ltd., Beijing, China), transformed into Trans1-T1 Phage Resistant competent cells, plated on agar containing ampicillin, and X-gal and white colonies were selected for further analysis. Following the expansion of the selected bacterial colonies, plasmid DNA was extracted by alkaline lysis (5). Restriction enzyme digests were used to validate successful recombination with final confirmation provided by sequencing. The sequencing results for each plasmid were compared with the NCBI sequences using the BLAST website. The five RIZ amplicons were digested from plasmids containing the correct insert and ligated into the eukaryotic pcDNA3.1(+) expression vector as described in Fig 3. Insertion was verified by restriction enzyme digestion and sequencing.

### Transfection of TE13 cells with pcDNA3.1(+)/RIZ1

The TE13 cells were seeded in six-well culture plates at a density of 2×10^5^ in a volume of 2 ml media, incubated at 37°C and allowed to reach a confluence of 90–95%. After 24 h, the media were replaced with complete serum-free RPMI-1640 or antibiotics-free medium in preparation for transfection. Ultra-pure pcDNA3.1(+)/RIZ1 plasmid DNA was extracted using the HighPure Mini Plasmid kit (Tiangen Biotech Co., Ltd.). A liposome-mediated method (6) was used to transfect the TE13 cells with the pcDNA3.1(+)/RIZ1 plasmid. Empty pcDNA3.1(+) plasmid and untransfected cells were used as negative controls. Following 6 h of incubation with media containing the recombinant plasmid and transfection reagents, the media were replaced with RPMI-1640, 10% FBS and antibiotics-free medium. The transfected cells were incubated for 48 h and harvested for further analysis.

### Quantitative PCR (qPCR) for RIZ1 mRNA

The cDNA from 28 paired human ESCC tissues, matched adjacent non-cancerous tissues and the TE13 cells were amplified using SYBR Premix Ex Taq™ (Takara). The primer (10 μM) sets that were used were as follows: Forward: 5′-TCTGCTGTTGACAAGACCC-3′ and reverse: 5′-GCATCAATGCACATCCATC-3′ for RIZ1; and forward: 5′-GAAGGTGAAGGTCGGAGTC-3′ and reverse: 5′-GGGTGGAATCATATTGGAAC-3′ for glyceraldehyde 3-phosphate dehydrogenase. The reactions were performed using a LightCycler (Roche Diagnostics, Mannheim, Germany) qPCR system according to the manufacturer’s instructions. Briefly, the reaction involved an initial denaturation step at 94°C for 5 min followed by 45 cycles of denaturation at 95°C for 5 sec, annealing at 59°C for 20 sec and extension at 72°C for 10 sec, followed by the generation of thermal melting curves. Each sample was run in triplicate for each gene.

### Western blotting

The PcDNA3.1(+)/RIZ1-transfected TE13 cells were homogenized in RIPA buffer (50 mM Tris-HCl, pH 7.4; 150 mM NaCl; 1% Nonidet P-40; 0.5% sodium deoxycholate; 0.1% SDS; 1 mM EDTA; 1 mM PMSF; 1 mg/ml aprotinin) and the protein concentrations were determined using the bicinchoninic acid protein assay kit (Pierce Biotechnology, Inc., Rockford, IL, USA). The cell lysates (30 μg) were separated by 8% SDS-PAGE, transferred to nitrocellulose membranes (Amersham Biosciences, Piscataway, NJ, USA) and immunoblotted with the indicated antibodies. All the antibodies were purchased from Abcam (Cambridge, UK), including the RIZ1 and the β-actin primary antibody and the secondary antibody goat anti-mouse. The bands were visualized using the PowerLook scanner (UMAX, Taipei, Taiwan) and quantified with ImageQuant software. The relative expression of RIZ1 was calculated as the gray value for RIZ1 divided by the gray value for β-actin. The TE13-untransfected and empty vector transfected cells were used as negative controls.

### Flow cytometric analysis

To determine the effect of overexpressing RIZ1 on apoptosis, 2×10^5^ TE13 cells were seeded in six-well plates and allowed to attach for 12 h. Cell cycle synchronization was achieved by serum starvation in serum-free RPMI-1640 media for 24 h. The cells were subsequently transfected with pcDNA3.1(+)/RIZ1 and harvested after 24 h. The cells were fixed in 70% ice-cold ethanol overnight, treated with DNase-free Ribonuclease (Takara), stained with propidium iodide (Sigma-Aldrich, St. Louis, MO, USA) and subjected to analysis using a FACSAria™ (Becton-Dickinson, Franklin Lakes, NJ, USA). The data were analyzed using ModFit LT software (Verity Software House, Topsham, ME, USA). The TE13-untransfected and empty vector transfected cells were used as controls.

### Treatment with 5-aza-CdR

The TE13 cells were seeded at a density of 2×10^5^ in six-well plates and treated with 10 μM DNMT inhibitor, 5-aza-CdR (Sigma-Aldrich), for 24–72 h. The drug was refreshed daily. The 5-aza-CdR was removed and the cells were subsequently incubated for 120 h.

### Statistical analysis

Statistical analysis was performed using SPSS 18.0 (SPSS, Inc., Chicago, IL, USA). The data are presented as the mean ± standard deviation. The qPCR results are presented as 2^−averageΔΔCT^ × 100%. T-tests and one-way ANOVA were used to analyze parametric data. The statistical analysis of the group comparisons involved one-way ANOVA and the χ^2^ test was used to compare enumerated data; P<0.05 was considered to indicate a statistically significant difference.

## Results

### Re-expression of RIZ1 by transfection of pcDNA3.1(+)/RIZ1

**Figure 1:**
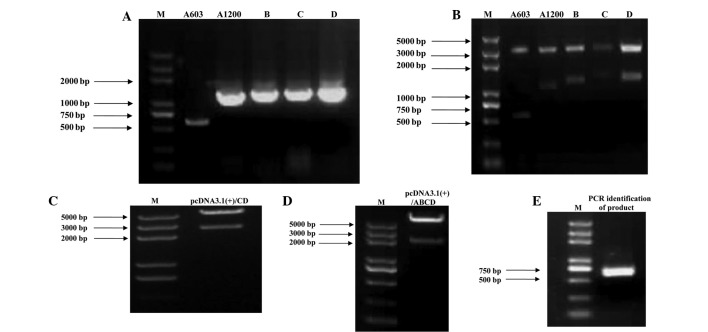
(A) qPCR results for the amplification of each RIZ1 amplicon. The size of the protein coding region was 5,157 bp positioned between 857 and 6,013 bp. Due to the size of the amplicon, the open reading frame was divided into five sections, designated A603, A1200, B, C and D. (B) Amplification and ligation of the RIZ1 amplicons into the pGEM-T vector. The five amplicons were each inserted separately into the pGEM-T vector. Restriction enzyme digestion verified that the digested products were of the expected size. (C–E) Generation of the recombinant pcDNA3.1(+)/RIZ1 construct. (C) The C and D segments were inserted into the pcDNA3.1(+) vector and verified by restriction enzyme digestion. The pcDNA3.1(+)/CD band of 2,583 bp was consistent with the theoretical results. (D) The A1200 and B gene segments were ligated into pcDNA3.1(+)/CD and verified by restriction enzyme digestion. The pcDNA3.1(+)/ABCD band of 2,000 bp was consistent with the theoretical results. (E) The A603 (PR domain) segment was inserted into pcDNA3.1(+)/ABCD and verified by restriction enzyme digestion. The 720 bp band was consistent with the theoretical results. qPCR, quantitative polymerase chain reaction; RIZ1, retinoblastoma protein-interacting zinc finger gene 1; M, marker.

**Figure 2:**
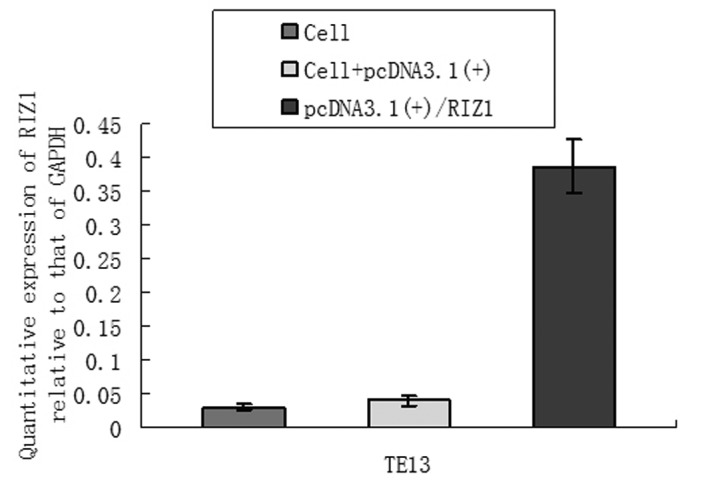
qPCR analysis of RIZ1 mRNA expression in TE13 cells transfected with pcDNA3.1(+)/RIZ1. RIZ1 mRNA expression was normalized to that of GAPDH. The difference was statistically insignificant between the empty vector and untransfected controls groups (P>0.05), whereas the deviation between the comparison groups and the pcDNA3.1(+)/RIZ1-transfected group was statistically significant (P<0.01). qPCR, quantitative polymerase chain reaction; RIZ1*,* retinoblastoma protein-interacting zinc finger gene 1; GAPDH, glyceraldehyde 3-phosphate dehydrogenase.

**Figure 3:**
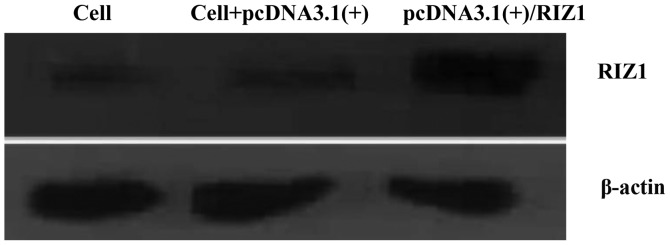
Western blot analysis of RIZ1 protein expression in TE13 cells transfected with pcDNA3.1(+)/RIZ1. β-actin was used as a loading control. Untransfected TE13 cells were used as a control. Significantly higher RIZ1 protein expression (P<0.01) was observed in the pcDNA3.1(+)/RIZ1-transfected cells, compared with the empty vector and untransfected controls. RIZ1, retinoblastoma protein-interacting zinc finger gene 1.

**Figure 4:**
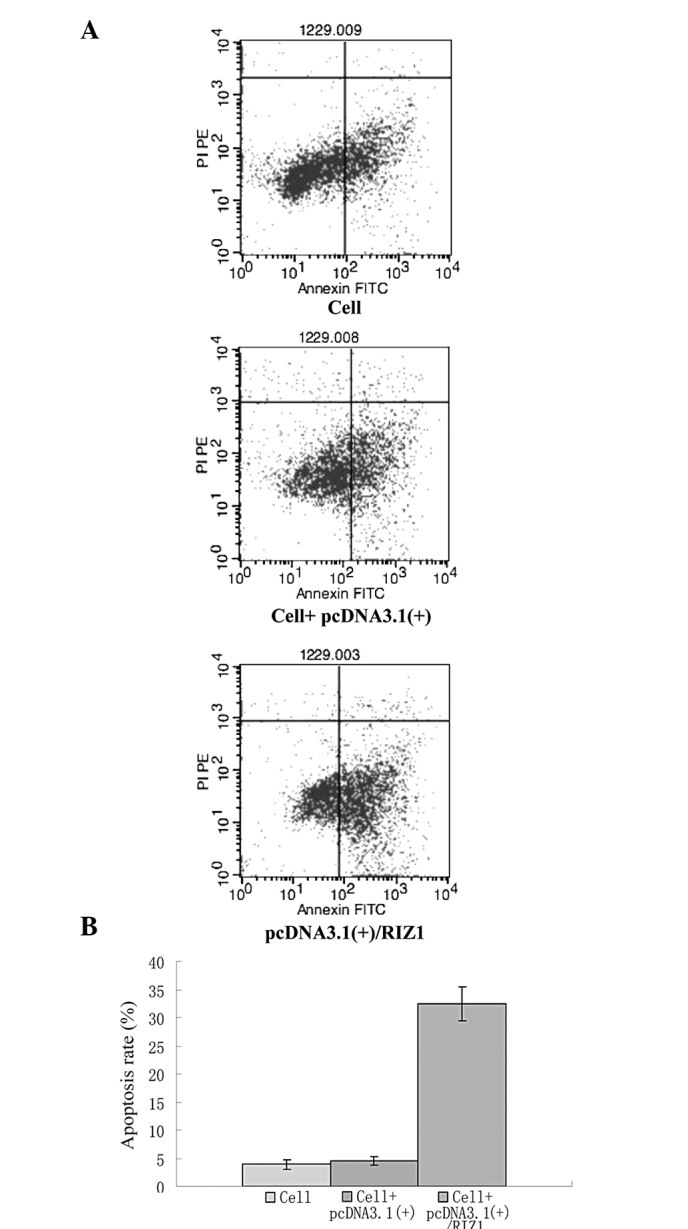
Flow cytometric analysis of the rate of apoptosis in the TE13 cells that were transfected with pcDNA3.1(+)/RIZ1. (A) Representative flow cytometric plots. (B) The rate of apoptosis was significantly higher in the pcDNA3.1(+)/RIZ1-transfected cells (P<0.01) than in the TE13 cells that were transfected with the empty pcDNA3.1(+) vector or the untransfected cells. RIZ1, retinoblastoma protein-interacting zinc finger gene 1; PI, propidium iodide; PE, phycoerythrein; FITC, fluorescein isothiocyanate.

In order to re-express RIZ1, a recombinant plasmid was generated to enable the ectopic overexpression of RIZ1 in TE13 cells. Due to the size of the protein coding region of the RIZ1 gene, the target was divided into five amplicons, A603, A1200, B, C and D. RNA was isolated, reverse transcribed into cDNA and amplified by PCR. Each of the five amplicons were subsequently ligated into the T Trans1-T1 Phage Resistant vector and used to transform the competent bacterial cells. Blue-white screening and ampicillin selection was used to select the potential positive colonies. Following the expansion of the colonies in liquid culture, the plasmid DNA was extracted by alkaline lysis. Successful recombination was verified by restriction enzyme digestion and sequencing. The five amplicons of the RIZ1 fragment were then ligated into the eukaryotic pcDNA3.1(+) expression vector. The RIZ1 fragment was successfully inserted into the pcDNA3.1(+) plasmid to produce recombinant pcDNA3.1(+)/RIZ1, with each amplicon in the correct order (Fig. 1). qPCR and western blot analysis demonstrated an increase in RIZ1 mRNA and protein expression in the TE13 cells that were transfected with the recombinant plasmid (Figs. 2 and 3). An analysis of the cell cycle using flow cytometry demonstrated that the rate of apoptosis increased in the TE13 cells subsequent to the transfection of the pcDNA3.1(+)/RIZ1 plasmid (Fig. 4).

### Re-expression of RIZ1 by 5-aza-CdR treatment

**Figure 5:**
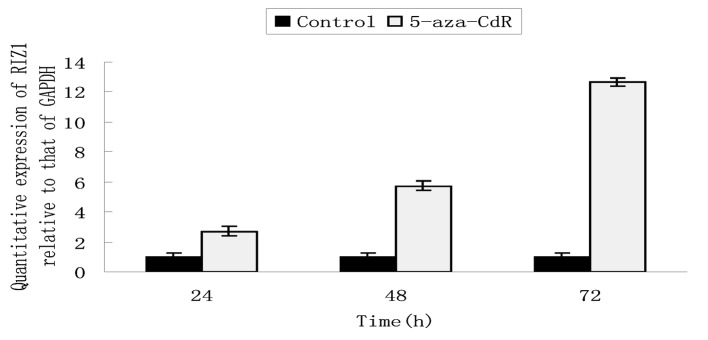
qPCR analysis of RIZ1 mRNA expression in the TE13 cells that were treated with 5-aza-CdR. RIZ1 mRNA level was expressed relative to that of GAPDH and calculated as 2^−averageΔΔCT^ × 100%. RIZ1 mRNA expression increased the longer the cells were treated with the drug (P<0.05). qPCR, quantitative polymerase chain reaction; RIZ1*,* retinoblastoma protein-interacting zinc finger gene 1; 5-aza-CdR, 5-aza-2′-deoxycytidine; GAPDH, glyceraldehyde 3-phosphate dehydrogenase.

**Figure 6:**
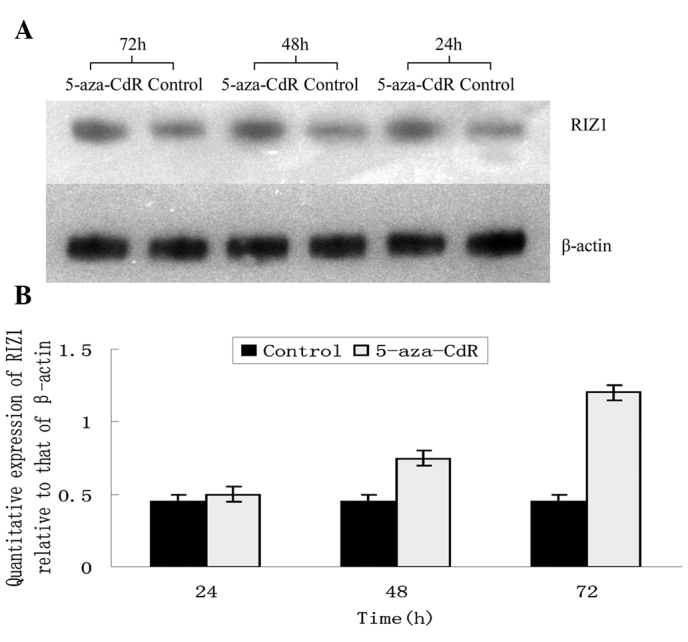
Western blotting of RIZ1 protein expression in the TE13 cells that were treated with 5-aza-CdR. (A) TE13 cells were treated with 5-aza-CdR for 24, 48 or 72 h and subjected to western blotting. (B) Quantification of RIZ1 protein expression. 5-aza-CdR significantly increased RIZ1 protein expression compared with that of the control cells in 48 or 72 h (P<0.01) exclusive of the 24 h group. Treatment with 5-aza-CdR for 48 or 72 h significantly increased RIZ1 protein expression compared with that in the 24 h group (P<0.01). RIZ1, retinoblastoma protein-interacting zinc finger gene 1; 5-aza-CdR, 5-aza-2′-deoxycytidine.

**Figure 7:**
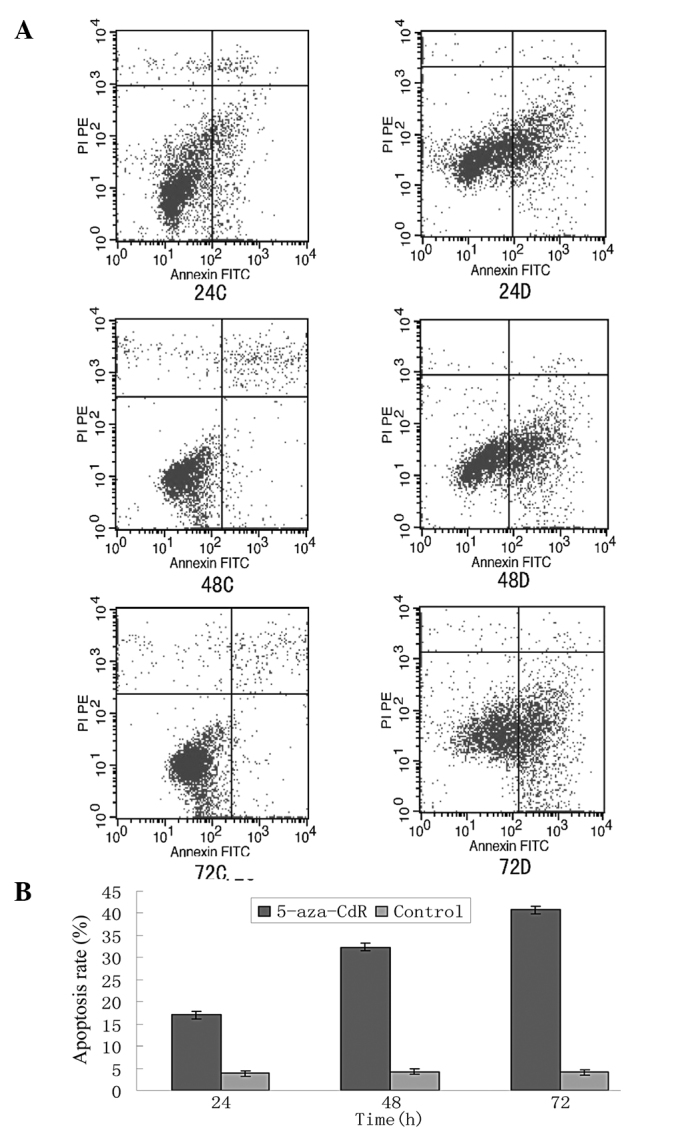
Flow cytometric analysis of the rate of apoptosis in the TE13 cells that were treated with 5-aza-CdR. (A) Representative flow cytometric plots. (B) Quantification of the apoptotic index. The rate of apoptosis increased with the time of exposure to 5-aza-CdR (P<0.01). 5-aza-CdR, 5-aza-2′-deoxycytidine; PI, propidium iodide; PE, phycoerythrein; FITC, fluorescein isothiocyanate.

The promoter of the RIZ1 gene in the TE13 cells, which expressed low levels of RIZ1, was observed to be methylated. The loss of this methylation was hypothesized to result in the re-expression of RIZ1 (4). Therefore, the TE13 cells were treated with 10 μM DNMT inhibitor, 5-aza-CdR. qPCR and western blotting demonstrated that the mRNA and protein levels of RIZ1 were significantly higher in the 5-aza-CdR group compared with the control cells at every time point (Figs. 5 and 6). 5-aza-CdR increased the levels of RIZ1 mRNA and protein expression in a time-dependent manner. Furthermore, the rate of apoptosis in each 5-aza-CdR group increased following the treatment with 5-aza-CdR. Additionally, 5-aza-CdR increased the rate of apoptosis in a time-dependent manner between 24 and 72 h (Fig. 7).

## Discussion

RIZ was first isolated by Buyse *et al*(7) using retinoblastoma (Rb) probes, whilst performing a functional screen for Rb, and it was observed that RIZ was able to interact with Rb. Fluorescent *in situ* hybridization demonstrated that the *RIZ* gene is located on human chromosome 1p36. There are two variants of RIZ, RIZ1 and RIZ2, due to the presence of two alternative initial locations (8). The sequences of RIZ1 and RIZ2 are identical, with the exception of the presence of a PR domain in RIZ2. The PR domain in RIZ2 is termed the PRDI-BF1-RIZ1 homology region and contains >100 amino acids. The function of this domain is to act as a protein-binding-interface, mediate protein-protein interactions, stabilize chromosomal structures and, therefore, regulate gene expression (9). Gene families that contain a PR domain contribute to tumorigenesis via a unique mechanism. The absence or presence of a PR domain results in the differential expression of proteins at an early stage of tumorigenesis, which provides a mechanism for tumor initiation (10). Studies have shown that RIZ1 is able to inhibit tumor development and is considered to be a significant tumor suppressor gene. Furthermore, as RIZ1 acts in combination with Rb, which induces the arrest of tumor cells in the G_2_/M phase leading to cell death, the combined re-expression of RIZ1 and Rb may halt tumor growth (11,12).

As a novel tumor suppressor, RIZ1 has been analyzed in numerous studies, which have attempted to understand the mechanism by which the gene is inactivated in cancer cells. Genetic and epigenetic changes are believed to be responsible (13). From a genetic perspective, RIZ1 may be deactivated by chromosomal instability and microsatellite instability, as well as frameshift mutations, point mutations and heterozygote deficiency (14). From an epigenetic perspective, the deactivation of RIZ1 may occur due to promoter methylation and histone acetylation. Changes to chromosome 1p36, on which RIZ1 is located, are also associated with numerous types of cancer, including breast cancer, ovarian cancer, liver cancer, colorectal cancer, chronic myeloid leukemia, melanoma, chromaffin tumor and neuroblastoma (15). In all these tumor types, the tumor tissue samples and cancer cell lines display low expression levels or deficiency of RIZ1. Furthermore, our previous study demonstrated that esophageal cancer tissues expressed lower levels of RIZ1 mRNA and protein compared with the normal tissues (3). Taken together, this evidence indicates that the deactivation of the RIZ1 tumor suppressor gene may be significant in the progression of esophageal cancer.

Treatment for ESCC may include surgery, radiotherapy and chemotherapy. However, the efficacy of such treatment for later stage disease is unsatisfactory. Furthermore, the side effects due to radiotherapy and chemotherapy, including the depletion of bone marrow, gastrointestinal toxicity, immunological suppression and liver or kidney injury, are far from negligible (16). At present, there are a number of noteworthy therapies that are being developed, including targeted drugs, conjugated drugs and nanoparticles. Gene therapy is an active area of research and holds much promise for the future of anti-cancer therapy (17). The development of a malignant tumor is caused by the inactivation of tumor suppressor genes and the abnormal activation of oncogenes. The main techniques that are being developed for the treatment of cancer by gene therapy include immune, causal, suicide and auxiliary gene therapy (18). The re-expression of a specified gene in a target cell is a new approach. However, if successfully applied, such approaches may also be useful for the management of a number of other diseases, in addition to cancer (19). Despite much progress being made in the technology of the vehicles that are used to deliver target genes to the appropriate cells, numerous vehicles have significant shortcomings and extensive research is required prior to the successful use of gene therapy in the clinic (20).

The pcDNA3.1(+) vector is an efficient eukaryotic expression vector. Transcription of the inserted sequence is controlled by the human cytomegalovirus promoter, whilst there is a transcription termination signal downstream (7,21,22). Compared with the viral vectors that are used for gene therapy, pcDNA3.1(+) does not stimulate an immune response, has no latent toxic side effects and does not require the dissemination of live viruses. Using a liposome-mediated transfection method, foreign genes may easily be inserted into target cells. Therefore, the pcDNA3.1(+) vector was selected to create pcDNA3.1(+)/RIZ1 for transfection into the eukaryotic cells.

RIZ1 is a tumor suppressor gene that prevents the progression of esophageal carcinoma. In the present study, the eukaryotic pcDNA3.1(+)/RIZ1 expression plasmid was used to transfect human squamous esophageal carcinoma TE13 cells, in order to re-express RIZ1. Furthermore, the re-expression of RIZ1 was attempted using the DNMT inhibitor, 5-aza-CdR, to reverse the methylation of the RIZ1 promoter in the TE13 cells. The DNMT inhibitor, 5-aza-CdR, has been used in the clinic for the treatment of certain solid tumors and hematological diseases, including myelodysplastic syndrome and acute myeloid leukemia (3,4). New therapeutic approaches for the treatment of esophageal cancer may be identified through further research into the epigenetic status of genes and the appropriate application of 5-aza-CdR.

The present study demonstrated that the transfection of pcDNA3.1(+)/RIZ1 or the application of 5-aza-CdR increased the expression of RIZ1 and effectively increased the rate of apoptosis in TE13 cells. This raises the possibility that the re-expression of RIZ1 may induce apoptosis in malignant esophageal cancer cells. Furthermore, the experiments have led to the development of a cell line in which RIZ1 is overexpressed and the detailed biological characterization of this cell line will follow. Future studies will focus on *in vivo* models in order to establish new methods to combat and potentially cure esophageal carcinoma using gene and cellular transplantation therapy. Additionally, further research on the mechanism of action of the RIZ1 tumor suppressor gene may lead to the development of new biomarkers for early diagnosis and prognostic evaluation in esophageal cancer.
